# Understanding the impact of RapidArc therapy delivery errors for prostate cancer

**DOI:** 10.1120/jacmp.v12i3.3409

**Published:** 2011-05-20

**Authors:** Mike Oliver, Karl Bush, Sergei Zavgorodni, Will Ansbacher, Wayne A. Beckham

**Affiliations:** ^1^ Department of Medical Physics British Columbia Cancer Agency Victoria British Columbia Canada

**Keywords:** RapidArc, volumetric‐modulated arc therapy, intensity‐modulated radiation therapy, MLC errors, gantry errors, MU errors

## Abstract

The purpose of this study is to simulate random and systematic RapidArc delivery errors for external beam prostate radiotherapy plans in order to determine the dose sensitivity for each error type. Ten prostate plans were created with a single 360° arc. The DICOM files for these treatment plans were then imported into an in‐house computer program that introduced delivery errors. Random and systematic gantry position (0.25°, 0.5°, 1°), monitor unit (MU) (1.25%, 2.5%, 5%), and multileaf collimator (MLC) position (0.5, 1, 2 mm) errors were introduced. The MLC errors were either random or one of three types of systematic errors, where the MLC banks moved in the same (MLC gaps remain unchanged) or opposing directions (increasing or decreasing the MLC gaps). The generalized equivalent uniform dose (gEUD) was calculated for the original plan and all treatment plans with errors introduced. The dose sensitivity for the cohort was calculated using linear regression for the gantry position, MU, and MLC position errors. Because there was a large amount of variability for systematic MLC position errors, the dose sensitivity of each plan was calculated and correlated with plan MU, mean MLC gap, and the percentage of MLC leaf gaps less than 1 and 2 cm for each individual plan. We found that random and systematic gantry position errors were relatively insignificant (< 0.1% gEUD change) for gantry errors up to 1°. Random MU errors were also insignificant, and systematic MU increases caused a systematic increase in gEUD. For MLC position errors, random MLC errors were relatively insignificant up to 2 mm as had been determined in previous IMRT studies. Systematic MLC shift errors caused a decrease of approximately −1% in the gEUD per mm. For systematic MLC gap open errors, the dose sensitivity was 8.2%/mm and for MLC gap close errors the dose sensitivity was −7.2%/mm. There was a large variability for MLC gap open/close errors for the ten RapidArc plans which correlated strongly with MU, mean gap width, and percentage of MLC gaps less than 1 or 2 cm. This study evaluates the magnitude of various simulated RapidArc delivery errors by calculating gEUED on various prostate plans.

PACS numbers: 87.55.x, 87.55.D, 87.55.de, 87.55.dk

## I. INTRODUCTION

Novel arc therapy algorithms are gaining prominence in radiation therapy due to similar plan quality as intensity‐modulated radiation therapy (IMRT) with more efficient delivery. The concept of intensity‐modulated arc therapy (IMAT) was first proposed by Yu in 1995 as an alternative to tomotherapy.^(1)^ Advancement in arc therapy optimization and delivery came when Earl introduced intensity‐modulated arc therapy‐based direct aperture optimization, followed by Otto's introduction of volumetric‐modulated arc therapy (VMAT).^(^ ^2^ ^,^ ^3^ ^)^ VMAT is a way to create arc therapy treatment plans that rival the plan quality of those that are created with IMRT, yet can be delivered in a few minutes. VMAT sparked a new‐found interest in arc therapy, and led vendors to implement arc therapy commercially, such as RapidArc (Varian Medical Systems, Palo Alto, CA, USA), VMAT (Elekta AB, Stockholm, Sweden) and SmartArc (Philips Medical Systems, Stockholm, Sweden).

Researchers are now performing VMAT treatment planning studies for brain, breast, head and neck, cervix, prostate, and other sites.^(^ ^4^ ^–^ ^11^ ^)^ Modern arc techniques such as RapidArc require that the multileaf collimator (MLC) leaves, gantry positions, and dose rate all change dynamically during delivery. These techniques are more complex than IMRT due to the dynamic nature of delivery and, therefore, require additional commissioning, machine‐specific quality assurance (QA), and patient‐specific QA.^(12)^

The focus of this study is to understand how random and systematic RapidArc delivery errors (in gantry positions, MU per control point or MLC positions) affect the calculated dose distributions for treatment of prostate cancer. An in‐house program introduced errors into the delivery parameters of prostate plans, and the effects of these errors were evaluated using gEUD.^(13)^ This is the first study to investigate the impact of RapidArc delivery errors. Previous studies have simulated IMRT delivery errors for gantry position errors and MLC positions errors; however, there have not been any studies that have investigated MU errors. Xing and colleagues^(14)^ investigated the effect of gantry position misalignment for two patients and determined that a 5° gantry position error resulted in a 1.5% decrease in the target dose or a 5.1% increase in the spinal cord dose. MLC position errors have been evaluated in simulations in numerous studies for IMRT: for step and shoot IMRT, Luo et al.^(15)^ investigated systematic MLC position errors in one of the MLC banks for eight prostate cancer patients, and found a strong linear correlation between MLC position error and mean target dose errors with a sensitivity of about 5%/mm error. Mu and Xia^(16)^ performed another step and shoot IMRT study of head and neck cancer treatment plans and determined that random MLC position errors had a small effect, but that systematic MLC errors that resulted in a closing or opening of the gap between MLC leaves were significant.

A large variability in dose sensitivity to systematic MLC position errors has been reported. A dose sensitivity of about 2.9%/mm was estimated for plans with less than 50 segments and 10%/mm for plans with greater than 100 segments.^(16)^ For dynamic (sliding window) MLC IMRT, Rangel and Dunscombe^(17)^ performed an analysis of random and systematic MLC position errors for prostate cancer and head and neck cancer. Their study determined that random MLC errors are relatively insignificant. However, for systematic MLC errors (open/close MLC gap), the dose sensitivity was 2.7%/mm for prostate and 5.6%/mm for head and neck cancer.

In the current study the dose sensitivity to RapidArc gantry errors, MU errors, and MLC errors is assessed for single arc prostate treatment plans.

## II. MATERIALS AND METHODS

### A. Patient cohort and planning parameters

Five prostate cancer datasets were selected for this study. Each dataset consists of a CT scan and contours of the prostate, planning target volume (PTV), bladder, and rectum. The PTV consisted of a 1 cm uniform expansion of the intact prostate. The prescription dose, 74 Gy in 37 fractions, as well as planning constraints, were taken from a previous VMAT planning study.^(10)^ The planning objectives for the organs at risk for the rectum were: V70 Gy < 5%, V40 Gy < 25%, V20 Gy < 50%, and for the bladder were: V40 Gy < 25% and V20 Gy < 50%. Plans were normalized post‐optimization such that 95% of the volume of the PTV received 74 Gy.

### B. RapidArc planning

For each patient, two treatment plans were created using RapidArc (Eclipse v8.6 BETA and v9.0BETA, Varian Medical Systems, Palo Alto, CA, USA). Dose distributions were calculated on $0.25\times 0.25\times 0.25\;{\text{cm}}^{3}$ grid. The first plan was created with lower MU values using Eclipse v8.6 BETA; the second was created with higher MU values using Eclipse v9.0 BETA, and correspondingly smaller mean MLC apertures. The optimization algorithm for Eclipse v9.0 BETA was modified and, as a result, produces plans with higher MUs. A single counterclockwise arc with a gantry range of 179.9° to 180.1° was used with the collimator set to 45°.^(18)^ Treatment plan optimization was initiated with the constraints, as noted in Section A above. Plans without any introduced errors are termed the “Baseline” plans, and were exported in DICOM format to allow modification of the delivery parameters. It is important to note that the resultant RapidArc plans are indexed to MU rather than time in the DICOM plan file.

### C. Modeling delivery errors for RapidArc plans

The DICOM plan file for each of the ten “Baseline” treatment plans was imported into an in‐house program written in MATLAB (MathWorks Inc., Natick, MA, USA) that modified the gantry positions, MU values or MLC leaf positions for each of the 177 control points within the RapidArc plans.

#### C.1 Gantry position errors

Systematic and random gantry position errors were introduced into each plan for error magnitudes of 0.25°, 0.5°, and 1°. Systematic errors in gantry position were simply an addition of the error magnitudes to the baseline gantry positions, while the random gantry position errors were introduced by sampling a Gaussian distribution with a standard deviation equal to the error magnitude. For plans with random error magnitudes of 1°, the gantry positions were modified after introducing the error to ensure that the sequence of all gantry positions remained counterclockwise throughout the arc.

#### C.2 MU errors

Systematic and random MU errors were also introduced into the treatment plans for error magnitudes of 1.25%, 2.5% and 5%. Systematic errors resulted in a constant percentage change in the MU increment for each control point within the plan, which is equal to incrementing the total MU for the plan. The random MU errors involved perturbing the MU increment for each control point within the arc by sampling a Gaussian distribution with a standard deviation equal to the error magnitude.

#### C.3 MLC errors

**Figure 1:**
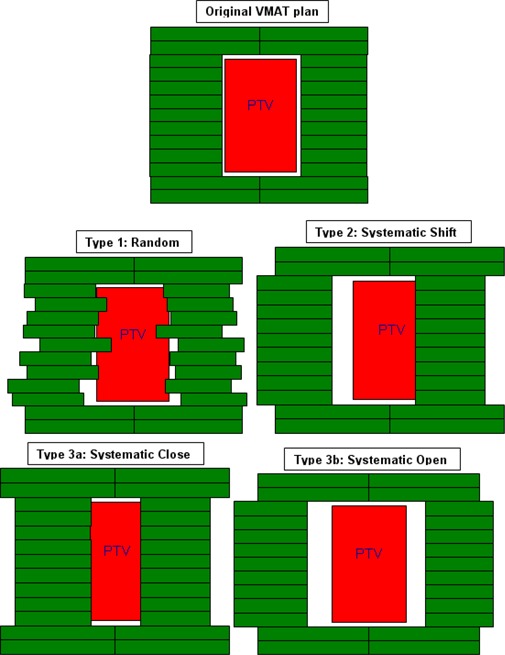
An example MLC shape which conforms to the PTV (red) for one control point of the arc for baseline plan (top) which is then modified with: random MLC positional errors (mid‐left), a systematic MLC shift (mid‐right), a systematic closing of the MLC leaves (bottom left), and a systematic opening of the MLC positions (bottom right).

Three types of systematic and one type of random MLC position errors were introduced into RapidArc plans for error magnitudes of 0.5, 1 and 2 cm. Systematic shift errors involved both MLC banks moved in the same direction by adding the error magnitude to each leaf position. “Close” errors involved both MLC banks being moved in opposite direction by the error magnitudes so that the MLC leaf gap is reduced. “Open” MLC errors involved both MLC banks being moved in opposite direction by the error magnitudes so that MLC leaf gap is increased. In addition, random MLC errors were also introduced by adding or subtracting random errors determined by sampling a Gaussian function with error magnitude equal to the standard deviation. If any error introduction resulted in a negative leaf gap, that gap was set to zero. A graphic demonstrating the different types of MLC errors is shown in Fig. 1.

### D. Dose calculation for error‐induced plans

All error‐induced treatment plans were imported back into Eclipse for dose recalculation. In several instances the treatment plans invoked warning messages within the planning system indicating that the plan had MLC or gantry speeds that exceeded tolerances. As the plans were not actually delivered, it was not possible to determine whether the warnings would have prevented treatment.

### E. gEUD analysis

Dose volume histograms for all plans were exported from the treatment planning system, and the generalized equivalent uniform (gEUD) dose was calculated for each structure from each plan.^(12)^ The “a” parameters that were used to calculate the gEUD: −10 for the PTV and 6 for both the bladder and rectum, were taken from a previous study.^(19)^ Plans were analyzed to identify the change in gEUD in percent for each error magnitude using Eq. (1):

(1) $$\Delta gEUD_{x}=\frac{gEUD_{x}-gEUD_{Baseline}}{gEUD_{Baseline}}x100\%$$

where the subscript “x” indicates plan being evaluated.

In addition, the ΔgEUD values for random and systematic error types were fit using linear regression for each of the types of errors for the entire group. The slope parameter from the linear regression fit for the group was used to determine the dose sensitivity to gantry, MU, and MLC position errors.

### F. Identifying sources of variability to MLC positions errors

It was postulated that the distance between opposing leaf pairs may be a predictor of MLC error sensitivity.^(17)^ In this section, the sources of variability for dose sensitivity to MLC errors are determined. Instead of investigating the dose sensitivity for a group of treatment plans, each treatment plan's dose sensitivity to MLC errors was calculated in units of ΔgEUD(%)/mm. These dose sensitivities were then plotted as a function of the total number of MU in the plan, the “mean gap” (the mean distance between opposing MLC leaves), the percentage of leaf gaps less than 1 cm, and the percentage of leaf gaps less than 2 cm. The dose sensitivity for each individual plan was then determined using linear regression.

## III. RESULTS

**Figure 2:**
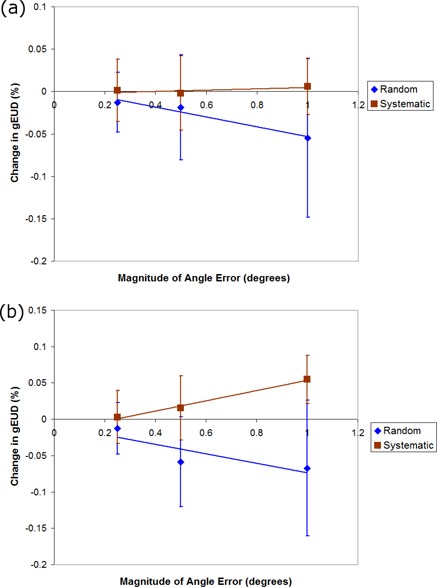
Two plots of the change in gEUD (%) as a function of gantry angle error for (a) PTV and (b) rectum for random (diamond) and systematic (square) gantry position errors.

**Figure 3:**
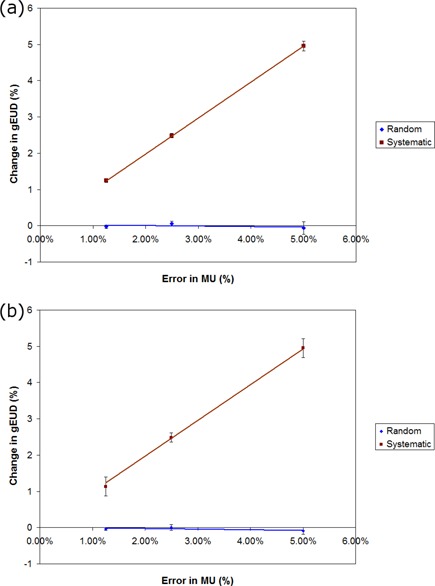
Two plots of the change in gEUD (%) as a function of MU error (%) for (a) PTV and (b) rectum for random (diamond) and systematic (square) gantry position errors.

**Figure 4:**
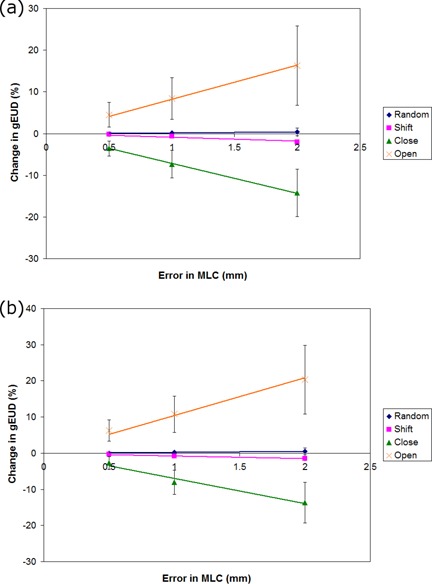
A plot of the change in gEUD in percent as a function of MLC errors for random (diamond), systematic shift (square), systematic MLC closing (triangle), and systematic MLC opening (X) for (a) the PTV and (b) rectum.

Figures 2 to 4 show the gEUD variation as a function of the modeled errors for PTV and rectum. Data for the bladder are not included in these figures because the values are similar to those for rectum. The mean MU values for the plans created with Eclipse v8.6BETA were 712±190 MU, and for plans created using Eclipse v9.0 BETA the mean MU values were 1388±348 MU.

### A. Gantry errors

The results for gantry position errors for the PTV and rectum are included in Fig. 2 for all ten treatment plans. In this study, random and systematic deviations in the gantry positions (up to 1°) had a change in gEUD of less than 0.1% for the PTV and rectum, as shown in Fig. 2.

### B. MU errors

The results of MU errors for the PTV and rectum are shown in Fig. 3, for all ten treatment plans. For random MU errors, the gEUD change for the PTV and rectum was less than 0.1% for 5% random changes in the MU for each control point. For systematic increases in the MU, a corresponding linear increase in gEUD was observed for both PTV and rectum. Linear regression values for percent gEUD increase as a function of systematic MU increases for the PTV were y=0.992x with $R^{2}=1$, and for the rectum the values were y=0.987x with $R^{2}=0.999$. As would be expected, there is a very strong correlation between the MU delivered and the gEUD.

### C. MLC position errors

The results for MLC errors are summarized in Fig. 4 for all ten treatment plans for the PTV and the rectum. Based on the results shown in Fig. 4, it is apparent that the least significant MLC error type is random, followed by a systematic shift in the MLC positions. The most sensitive MLC errors were observed when closing or opening the MLC leaves, which demonstrated high‐dose sensitivities.

### D. Linear regression for gantry, MU, and MLC position errors

The gEUD was found to have a near‐linear correlation with MLC position and gantry rotation, as well as MU errors. Linear regression fit parameters and $R^{2}$ values for all of the error types are included in Table 1.

**Table 1:** A complete listing of the dose sensitivity in units of %/degree, %/%MU, and %/mm along with correlation coefficient values for random and systematic gantry position errors, MU errors, and MLC position errors for the PTV, bladder, and rectum. X signifies that the result is not applicable.

		*Gantry*		*MU*		*MLC*	
		*Dose Sensitivity (%/°)*	$R^{2}$	*Dose Sensitivity (%/%)*	$R^{2}$	*Dose Sensitivity (%/mm)*	$R^{2}$
PTV	Random	−0.050	0.982	−0.005	0.136	0.191	0.966
	Systematic	0.004	0.938	0.992	0.999	−0.956	0.980
	Systematic Close	X	X	X	X	−7.160	1.000
	Systematic Open	X	X	X	X	8.183	1.000
Bladder	Random	−0.080	1.000	−0.005	0.088	0.387	0.992
	Systematic	−0.030	0.882	0.987	1.000	2.165	1.000
	Systematic Close	X	X	X	X	−6.459	0.990
	Systematic Open	X	X	X	X	10.897	0.999
Rectum	Random	−0.080	0.947	−0.014	0.762	0.261	1.000
	Systematic	0.050	0.965	0.991	1.000	−0.777	1.000
	Systematic Close	X	X	X	X	−7.067	0.995
	Systematic Open	X	X	X	X	10.310	0.999

The PTV exhibited a dose sensitivity of −0.05%/degree for random gantry errors, 0.004%/ degree for systematic gantry errors, −0.005%/%MU for random MU errors, and 0.992% gEUD increase per percentage increase in MU.

For random MLC errors, the gEUD sensitivity for the PTV was 0.2% per mm of standard deviation (SD), for the bladder it was 0.4% per mm of SD, and for the rectum 0.3% per mm of SD. For systematic shift errors in the MLC, the gEUD sensitivity for the PTV was −1.0%/mm, for the bladder 2.2%/mm, and for the rectum −0.8%/mm. The largest changes in gEUD sensitivity for MLC errors were systematic MLC open errors, which were 8.2%/mm, 10.9%/mm, and 10.3%/mm for the PTV, bladder, and rectum, respectively. The corresponding results for the systematic MLC close errors were −7.2%/mm, −6.5%/mm, and −7.1%/mm for the PTV, rectum, and bladder, respectively. In order to convert these values to MLC gap errors, the numbers should be divided by 2 to reflect the fact that both leaves were moved in or out in this analysis.

### E. Sources of variability in dose sensitivity to MLC errors

**Figure 5:**
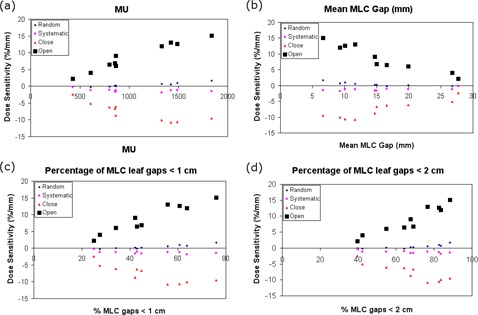
A set of graphs showing the dose sensitivity as a function of the number of MU for the PTV in: (a) the plan, (b) the mean MLC gap, (c) the percentage of MLC gaps that are less than 1 cm, and (d) the percentage of MLC gaps that are less than 2 cm. Included are random MLC errors (diamond), systematic MLC shift errors (small square), MLC gap close errors (triangle) and MLC gap open errors (big square).

The values presented in Table 1 provide a general guide to determine the dose sensitivity of various delivery errors for RapidArc. For MLC errors, however, there is a large variability in the dose sensitivity within the group, as can be seen from the magnitudes of the error bars in Fig. 4. We have plotted the dose sensitivity to MLC errors for the PTV of each plan with MU, mean gap width, the percentage of MLC gaps that are less than 1 cm, and the percentage of gaps that are less than 2 cm. The plots are shown in Fig. 5, along with coefficient of determination (R^2^) values included in Table 2. All sources of variability fit to a linear trend. In addition, the MLC open errors are fit by a linear trend with a high $R^{2}$ value (range: 0.90–0.95).

**Table 2:** A list of the coefficient of determination $(R^{2}$) values for a linear regression fit of the gEUD dose sensitivity for the PTV as a function of the total number of MU, the mean gap width, the percentage of MLC gaps less than 1 cm, and the percentage of MLC gaps less than 2 cm, for various types of MLC errors.

	*Random*	*Systematic Shift*	*Systematic Close*	*Systematic Open*
$R^{2}$ ‐ MU	0.88	0.26	0.77	0.95
$R^{2}$ ‐ Mean Gap Width	0.67	0.49	0.83	0.90
$R^{2}$ ‐ Percent of MLC gaps less than 1 cm	0.85	0.35	0.73	0.92
$R^{2}$ ‐ Percent of MLC gaps less than 2 cm	0.70	0.47	0.84	0.91

## IV. DISCUSSION

This study provides a framework for evaluating the potential clinical effects of simulated RapidArc machine delivery errors by calculating the gEUD changes for prostate plans. For gantry position errors, the gEUD sensitivity was found to be less than 0.1%/degree for all structures, which indicates that over the 0 to 1 degree range there is a very small change in gEUD. In addition, random errors in segment MU had an effect of less than 0.1% per %MU. Based on the authors' observation of gantry dynalog files following RapidArc delivery, these error ranges would cover virtually all gantry and MU deviations that could be expected. However, there were linear increases in gEUD corresponding to systematic increase in MU with a gEUD sensitivity of approximately 1% per percentage increase in MU for the PTV and similar values for the bladder and rectum.

For the MLC error data, it was determined that there was a large variability for each plan's dose sensitivity. For the second part of the analysis, the dose sensitivity for each plan was assessed by fitting the gEUD data for each plan using linear regression and determining the plan gEUD sensitivity to MLC errors. As shown in Fig. 5, these individual plan gEUD sensitivities exhibit correlations with four parameters: total number of MU in the plan, the mean gap width of MLC leaves, the percentage of MLC gaps that are less than 1 cm, and the percentage of MLC gaps that are less than 2 cm. The linear regression data listed in Table 2 shows a strong correlation for the systematic open and close MLC errors as a function of all variables. The implication of these results is that if a RapidArc plan is to be robust against MLC open/close delivery errors, then the total number of MUs should be minimized and the MLC gaps between leaves should be kept as large as possible without sacrificing plan quality. Based on previous studies, these conclusions should be equally applicable to IMRT.^(^ ^16^ ^,^ ^17^ ^)^

Results of this study would be slightly affected by the dose grid size (2.5 mm) chosen in the calculations. It is well recognized that the grid size affects the shape of the DVH and would somewhat affect the calculated EUD values. This effect is, however, small and does not compromise the results presented.

The RapidArc results presented in this study for MLC errors can now be compared against previous IMRT studies. As has been reported in other IMRT MLC errors studies, random MLC errors for both RapidArc and IMRT are practically negligible.^(^ ^16^ ^,^ ^17^ ^)^ The results for systematic open/close MLC errors in this study estimated a gEUD sensitivity of 10/7.7% per mm for RapidArc. These values can be compared against those obtained by Rangel and Dunscombe^(17)^ for gEUD sensitivity for prostate cancer which were reported to be 2.7%/mm (independent of open or close errors). We hypothesize that the discrepancies may be attributed to the differences in the percentage of MLC gaps < 2cm. In the current study, 67% of MLC gaps were found to be less than 2 cm for RapidArc (dose sensitivity =−7.2% or 8.2%), as compared to Rangel and Dunscombe's prostate data where 31% of MLC gaps were separated by 2 cm or less (dose sensitivity =2.7%) and 57% of leaf pairs were separated by 2 cm or less for head and neck (dose sensitivity=5.6%). So there is clearly a trend in which a higher proportion of leaves that are less than 2 cm results in an increase in dose sensitivity.

Previous arc therapy optimization studies have investigated perturbing gantry positions from their original planned positions, and demonstrated that the dosimetric error is minimal.^(^ ^20^ ^,^ ^21^ ^)^ It would be useful to determine at what point random and systematic gantry position errors become significant (i.e., cause more than 2% dose deviation for PTV), and whether this varies for disease sites.

The results regarding dose sensitivities obtained in this study can help guide patient‐specific QA efforts, as it has been shown that gantry position errors are relatively insignificant and, therefore, may not require as much attention as other subsystems. MLC position errors, where there are systematic increases/decreases in the MLC gaps, are found to a have very large impact on the gEUD and should be monitored carefully. In this study, we have assumed that RapidArc delivery errors may fall within a certain range. It would be useful to perform experimental studies to determine the typical long‐term accuracy and precision of the relevant parameters for the RapidArc delivery. Once these values are determined, this study can act as a template to translate these measured errors to better understand the clinical impacts of the RapidArc errors for a prostate cancer group. It is likely that these results can be translated to other disease sites; however, another study may be required to validate this hypothesis.

## V. CONCLUSIONS

In this study, an evaluation of the changes in gEUD caused by random and systematic errors (gantry, MU, and MLC) of RapidArc treatment plans has been performed for prostate radiotherapy. We found that of all parameters evaluated, systematic gap (open/close) errors had the most significant impact on gEUD, and its PTV dose sensitivity was about 8%/mm. There was a large variability for MLC gap open/close errors for the ten RapidArc plans. The sources of variability in gEUD sensitivity for individual RapidArc plans can be characterized either in terms of the total MU, mean MLC gap width, or percentage of MLC gaps less than 1 or 2 cm. In order to create plans that are robust against MLC errors, the total MU should be minimized and the MLC gaps increased as much as possible without sacrificing plan quality.

## ACKNOWLEDGMENTS

This work was supported in part by a grant from Varian Medical Systems.
